# Physiological, Behavioral, and Scientific Impact of Different Fluid Control Protocols in the Rhesus Macaque (*Macaca mulatta*)

**DOI:** 10.1523/ENEURO.0195-16.2016

**Published:** 2016-09-22

**Authors:** Helen Gray, Henri Bertrand, Claire Mindus, Paul Flecknell, Candy Rowe, Alexander Thiele

**Affiliations:** 1 Institute of Neuroscience, Newcastle University, Newcastle upon Tyne NE2 4HH, United Kingdom; 2 Comparative Biology Centre, Newcastle University, Newcastle upon Tyne NE1 7RU, United Kingdom

**Keywords:** motivation, nonhuman primate, welfare

## Abstract

Rhesus macaques are an important model in behavioral neuroscience due to their advanced cognitive abilities. To motivate animals to engage in complex tasks, fluid rewards, in conjunction with fluid control protocols, are often used. The impact of these protocols on animal welfare is controversial. We compared two fluid control protocols against a protocol providing free access to water and evaluated the impacts on physiological states of hydration, behavioral measures of welfare, and scientific output. Blood physiology did not significantly differ between any of the protocols, and urine measures were indicative of well functioning, healthy kidneys. Changes in behaviors were limited, the main one being an increase in motivation to drink on the stricter fluid control protocol, and improved task performance early in the week. Overall, fluid control protocols had little measurable impact on the welfare of rhesus macaques while ensuring that scientific data of high quality could be obtained.

## Significance Statement

This study demonstrates that two fluid control protocols caused no detrimental effects to the physiology of rhesus macaques. Behavioral impacts were limited, and laboratory performance improved during protocols with stricter controls. Overall, fluid control has much less of an impact than widely proposed.

## Introduction

Nonhuman primates are a widely used model in neuroscience, due to their close phylogenetic relationship to humans, and thus their relevance to understanding cognitive functions, brain disease, and aiding therapy (Roelfsema and Treue, 2014). Primate-specific behavioral and anatomical features include high manual dexterity, behavioral flexibility (Newsome and Stein-Aviles, 1999), highly evolved visual cortical specialization (Felleman and Van Essen, 1991), a granular prefrontal cortex (Preuss, 1995), and neuropharmacological specializations (Disney et al., 2006).

Cognitive and neural processes in awake, behaving primates are often investigated by means of electrophysiological and neural imaging methods. These require a high number of consecutively performed trials to achieve adequate data quality, statistical reliability, and sample sizes suitable for complex model testing. To achieve adequate daily performance, laboratories often use fluid rewards as motivators for correct trial performance and restrict fluid access for the animals outside of the experiment (Desimone et al., 1992; Newsome and Stein-Aviles, 1999; Toth and Gardiner, 2000; Hage et al., 2014). Despite the widespread use of fluid control protocols, and the justification for their implementation, their use has been an issue of increasing contention for >20 years (Orlans, 1991; Desimone et al., 1992; Willems, 2009; Prescott et al., 2010; Westlund, 2012). Concerns voiced include potential dehydration (Rowland, 2007), weight loss (Prescott et al., 2010), and pain or distress (Willems, 2009). For an adequate assessment of fluid control on animal welfare, physiological and behavioral measures sensitive enough to capture relevant changes in welfare must be used.

There have been previous attempts to evaluate the use of these techniques on some aspects of animal welfare using physiological or behavioral measures. Yamada et al. (2010) found that increases in macaque blood osmolality (Osm), caused by fluid control, could be quickly restored over the course of a behavioral task, and that osmolality remained mostly stable across a 5 day (d) working week. More recently, Hage et al. (2014) failed to detect any changes in home cage behavior across a 12 d period of fluid control. Although both of these studies help to alleviate some concerns of fluid control protocols, it could be argued that they are too focused on one particular type of measure or are too short term to address concerns about the long-term impacts on welfare.

We investigated the validity of the concerns surrounding fluid control in a controlled, within-subject design experiment in four macaques used in electrophysiological studies over a 16 week period. During this time, all four animals experienced two different fluid control protocols, which are used in primate research (Prescott et al., 2010). The physiological and behavioral outcomes of these fluid controls were compared with baseline data taken when the monkeys had free access to water, and where possible, to a nonrestricted age- and sex-matched control group at the Centre for Macaques (CfM) UK breeding facility. We thus combine a suite of physiological and behavioral measures to assess the impact of longer-term (16 weeks) use of different protocols on animal welfare and performance in behavioral tasks.

## Materials and Methods

All experimental animal procedures complied with European Union Directive 2010 (2010 63 EU), the National Institutes of Health (*Guidelines for the Care and Use of Laboratory Animals*), the Society for Neurosciences Policies on the Use of Animals and Humans in Neuroscience Research, and the Animals (Scientific Procedures) Act 1986.

### Animals and husbandry

Subjects were four male rhesus macaques (*Macaca mulatta*) with ages ranging between 7 and 9 years, and weighing between 9 and 15 kg. The monkeys were used for electrophysiology and/or MRI studies and were experienced in the experimental setup and behavioral tasks. Subjects were each pair housed with another male (not recruited for this particular study), in cages of either 2.1 × 3.0 × 2.4 m or 2.3 × 2.45 × 2.4 m, in a facility where individuals could have visual and auditory contact with many monkeys in other cages. Toys were given on a rotational basis as an environmental enrichment. Dry food mix was added to shavings that covered the floor to encourage foraging, as a stimulant and reward (Chamove, 1989), as recommended by primate welfare guidelines (NC3Rs, 2006). Outside the context of this study, the diet was supplemented by the provision of fruits and vegetables once a week. However, to obtain full control of the fluid intake of the animals, no fruits or vegetables were provided for the duration of the study. Outside of experimental fluid control periods, water was freely available in the home cage, while water provision was tightly controlled during the fluid control periods (see details below). The facility had a 12 h light/dark cycle from 7:00 A.M. to 7:00 P.M., as well as natural light from ceiling windows. The temperature and humidity were ∼20°C and 24%, respectively.

During the study, monkeys underwent daily checks by a technician or veterinarian. In case of a health or welfare concern, technicians and the veterinarian checked the animal several times per day. Fur condition, feces, eyes, food intake, and activity levels were visually assessed. Monkeys were sedated annually to assess their general health.

### Fluid control protocols

Fluid control protocols need to be tailored to each individual animal to ensure maximum motivation with minimum welfare impact. For each animal, the volume of water consumed under free access conditions [free access intake (FAI)] was determined over a period of at least 5 d (not necessarily consecutive days). Following this, starting at a minimum of 70% FAI, the performance of the animal in the experimental setup was determined over at least 3 d. The minimum was then decreased as necessary (in steps of 10-15% of FAI) until the animal was sufficiently motivated to work for fluid rewards in order to obtain scientifically useful data (∼1000–1200 correct trials in a daily session). After each reduction, the work rate of the animal was assessed for at least 3 working days to determine the current levels of motivation and performance. Further decreases were implemented only if the current minimum was insufficient to achieve the required number of daily trials.

We assessed the following three different conditions: a control period of free access to water (hereafter “free access”); and both a 5 d and a 7 d fluid control protocol (hereafter called the “5 d protocol” and the “7 d protocol”). The 5 d protocol consisted of 5 d of fluid control with free access to water on days 6 and 7. This is the standard protocol implemented in our laboratory. The second protocol consisted of 7 d of fluid control, where animals had access to their individually established minimum every day, which they could exceed by means of their work rate during experimental weekdays, but not on days 6 and 7. The 7 d protocol was tested to investigate whether it could result in improved scientific output, and what the potential welfare implications would be. On a 5 d protocol, work rates following the 2 d of free access are normally too low to allow for electrophysiology recordings, leaving at least 1 d/week where the animal is fluid restricted and works on the cognitive task in the laboratory without usable data being collected. If a 7 d protocol was more effective at motivating animals to perform the task on a Monday, data collection could be quicker and periods of fluid control could be reduced. Given these potential benefits, it was important to be able to compare welfare measures between protocols as well as to a control period of free access.

On the 5 d protocol, the subjects received their minimum allowance amount either in the laboratory (Monday to Thursday) or in the home cage (Sunday). On Friday evenings and Saturdays, they were given free access to water in the home cage. On the 7 d protocol, the monkeys received their minimum fluid intake every day (Monday to Friday in the laboratory and Saturday and Sunday in the home cage), but were never given free access to water. Each protocol lasted for 4 weeks at a time and was repeated twice (a total of 16 weeks of study, 8 weeks for each protocol). The protocols were given either in a 5-7-5-7 d order (two monkeys) or a 7-5-7-5 d order (two monkeys). The monkeys were sampled for blood and urine on the last Friday morning of each protocol (detailed below). After sampling, they were given free access to water for Friday morning and afternoon before the next protocol began on Saturday.

Animals worked 5 d/week (Monday to Friday) throughout the experiment. Within a daily experimental session, the monkey was allowed to work for as much fluid as he wanted, but in situations where the minimum daily allowance was not earned during the task, the monkey was supplemented (to its established minimum) with water in the laboratory after the session had finished. Therefore, monkeys received at least their minimum fluid allowance every working day. Two monkeys were rewarded with blackcurrant juice (Ribena, Lucozade Ribena Suntory Ltd), one monkey was rewarded with water and one with diluted Coca-Cola (the Coca-Cola Company). For the 16 weeks of fluid restriction, monkeys were separated from their cage mates from Friday evening until Sunday afternoon. This was done to obtain accurate recordings of fluid intake for the monkey of interest and to ensure that the cage mate had adequate (unrestricted) access to water for that period.

Prior to the fluid control protocols, the monkeys experienced a control period of 12 d, during which they had free access to water, and during which behavioral and physiological measures were obtained. A second control period of 12 d was conducted 6 months after the completion of the fluid control protocols, and physiological measures were obtained again and used with those from the first control period for analysis.

### Tasks performed by the primates

For the duration of this study, each monkey was also involved in ongoing neuroscience experiments, in which they were performing tasks in relation to visually presented stimuli to obtain fluid rewards. Three subjects were engaged in covert top–down attentional tasks with individual trial times of 2000–4000 ms. The other monkey was performing a memory-guided saccade task, with individual trials taking up to 5000 ms. Experiments were performed in a dimly lit room. Monkeys were weighed daily before the start of the task, and then transferred between the housing unit and the laboratory using a custom-made trolley, onto which the primate chair was fitted. Performance in the laboratory was monitored via computer control; task performance (i.e., the number of correct trials performed by the monkey in their task) was recorded for each session. The criteria for determining when the monkey had stopped working (e.g., no consistent task engagement for >15 min) differed slightly between animals and experimenter, but they remained consistent for individual monkeys over the course of the study. When animals had stopped working according to these criteria, they were transferred back to the cage. Experimenters were blind to which fluid control protocol their animal was currently subject to.

### Physiological measures

Physiological measures of the hydration state were collected at the end of the free-access periods (i.e., two data points per animal, one prior to implementing the fluid control protocols, the other 6 months after) and on the last day of each 4 week block of the 5 and 7 d protocols (i.e., two data points per animal per protocol). Animals were sedated with ketamine (10 mg/kg, i.m.), and blood was collected from the saphenous vein for hematological and biochemical analysis. During the sedation following the control period, the bladder was located using ultrasound, and urine was extracted via cystocentesis. During the fluid control protocols, this was not possible because of the small size of the bladder; instead, urine was collected from the cage on the morning of sedation, when possible. Urine was collected at least once per fluid control protocol for each monkey.

To compare results to a relevant baseline, blood samples were also obtained from the CfM, the UK rhesus macaque breeding facility. Fourteen male monkeys, with ages ranging from 4 to 15 years and weighing between 9 and 16 kg, were sedated as described above and blood was collected from the femoral vein. The CfM monkeys received free access to water at all times and were group housed. Due to sampling procedures, it was not possible to obtain urine samples from the monkeys at CfM.

### Weight data

Animals were weighed on each weekday before being taken to the laboratory. This allowed for an evaluation of weight change over the course of a working week as well as a further assessment over the duration of a fluid control block (4 weeks). The dataset obtained was not complete, and the following number of weights was collected for each animal of a possible 76 d (38 d/protocol, as animals were not taken to the laboratory, and thus not weighed, on physiological sampling days): Monkey 1, 65 weights; Monkey 2, 75 weights; Monkey 3, 67 weights; Monkey 4, 74 weights.

### Behavioral measures

In order to assess the potential psychological impact of different fluid control protocols, behavioral measures were collected while monkeys were in their home cages. Behavior was recorded using cameras (Cube HD 1080, Y-cam) attached to the corridors of the primate housing facility, outside of each cage of interest. Data were collected three times per week, as follows: early week (Monday evenings and Tuesday mornings); late week (Thursday morning and evening); and Weekend (Saturday morning and evening). Using a range of days permitted the assessment of changes in behavior throughout the week. Morning recordings lasted from 7:00 to 9:00 A.M., and evening recordings lasted from 5:00 to 6:40 P.M. (to coincide with lighting times). These times reduced the number of personnel present in the primate facility, which could on its own affect animal behavior.

An ethogram was designed to capture behaviors that were potentially associated with changes in the welfare state (Table 1). Behaviors were sampled in one of two different ways. They were scored either every time they occurred in a video observation (hereafter called “continuous sampling”) or they were scored at a 30 s sample point (“scan sampling”). Continuous sampling was used for short or rare behaviors in order for them to be captured by the observation duration. Continuously sampled behaviors could be recorded either as “frequency” data or as “duration” data. Frequency data consisted of counts of behaviors, whereas duration data also included the length of time a behavior was performed. A pilot set of behavioral observations (∼100 h spread across all animals) was analyzed to assess whether the full length of the recordings was needed to accurately capture potential behavioral changes induced by different protocols, or whether subsampling was sufficient. Using paired *t* tests for each animal, no difference was found between analyzing the middle hour (7:30 to 8:30 A.M., 5:20 to 6:20 P.M.) and the full recording. The remaining observations and all analyses were therefore performed using the middle hour only to ensure that representative data were obtained, while keeping analysis times manageable. In total, 393 h of video material were observed and analyzed with the following distribution across animals: Monkey 1, 105 h; Monkey 2, 101 h; Monkey 3, 88 h; and Monkey 4, 99 h.

**Table 1:** Behavioral measures of welfare

Category	Behaviors	Description	Sampling	Frequency/duration
Inactive	Alert	Sitting/lying/standing stationary on any surface and looking at objects or individuals inside or outside of the cage	Scan	Frequency
	Not alert	Sitting/lying/standing stationary on any surface, eyes may be open or closed, not looking at objects or individuals inside or outside of the cage	Scan	Frequency
	Hunched	As for not alert, but sitting with head lower than the shoulders	Scan	Frequency
Foraging	Eating	Ingestion of items	Scan	Frequency
	Foraging	Searching for food or manipulation of food items or sources, without ingestion of food	Scan	Frequency
	Chewing	Chewing without any insertion of food into the mouth in the preceding 30 s	Scan	Frequency
Abnormal	Locomotor stereotypy	One or more completions of a repeated locomotor pattern, including any embedded behaviors	Scan	Frequency
	Other abnormal	Digit sucking, hair pulling, nail biting, rocking, head flicking, hand shake, any self-injurious behavior	Continuous	Duration
Nonsocial behaviors	Self-groom	Stroking, picking, or otherwise manipulating own body surface	Scan	Frequency
	Self-scratching	Scratching the skin vigorously with nails	Continuous	Duration
	Yawn	Open the mouth widely, teeth exposed, lips retracted without vocalisation	Continuous	Frequency
	Body shake	Dog-like body shake of whole body	Continuous	Frequency
	Eye rub	Rubbing the eye with a hand	Continuous	Duration
	Interact with physical environment–hands/feet	Swinging, pushing, manipulating any part of the cage or an enrichment with hands or feet without using mouth	Scan	Frequency
	Interact with physical environment–oral	Manipulating any part of the cage or an enrichment with mouth involved. Chewing/licking/biting any aspect of the cage or inanimate object in it	Scan	Frequency
Social behaviors	Allogroom–donor	Stroking, picking, or otherwise manipulating a cage mate's body surface	Scan	Frequency
	Allogroom–recipient	Being groomed by cage mate, following above descriptors	Scan	Frequency
	Aggression to cage mate	Open mouth stare, threat posture, chase, push, attack	Continuous	Duration
	Submissive to cage mate	Fear grimace, present, displacement of position in the cage	Continuous	Duration
	Dominance to cage mate	Displace the cage mate out of position	Continuous	Duration
	Affiliative	Lipsmack	Continuous	Duration
	Aggression directed outside cage	Open mouth threat, attack or threat postures directed outside of the cage (e.g., at the glass)	Continuous	Duration
	Play with cage mate	Nonaggressive high intensity interaction (chase, wrestle, tumble) with cage mate	Scan	Frequency
	Mounting	Mounting cage mate	Continuous	Duration
	Being mounted	Being mounted by cage mate	Continuous	Duration
Locomotion	Agitated locomotion	Moving between locations, often rapidly, with a stiff unrelaxed gait	Scan	Frequency
	Relaxed locomotion	Moving between locations with a relaxed gait	Scan	Frequency
Other	Other	Any behavior not listed above and noteworthy; describe form	Continuous	Duration

### Water bottle approach and consumption

In order to gauge the motivational drive to drink under the different fluid control protocols, “latency to drink” was measured on Saturday and Sunday mornings. If the motivation to drink was increased on a stricter fluid control protocol, we would expect that latency to approach the bottle would be quicker on the 7 d protocol than on the 5 d protocol, and that the volumes consumed would be higher. A water bottle containing either the minimum allowance, or 1 L of water (depending on the fluid control protocol) was attached to the home cage, and the latency to start drinking was recorded. In circumstances where the monkey began to drink before the bottle was fully attached to the cage, the latency was scored as <1 s and given a value of 0.5 s for analysis. As the volumes of water offered on Saturdays differed between the two protocols, an additional test was performed on Saturday morning, whereby the amount of fluid consumed in the first 5 min was also measured.

### Statistical methods

All analyses were performed using SPSS version 21 (IBM, 2012) and R (R Foundation for Statistical Computing, 2015). R software was used when a model was not available in SPSS, and the R packages used were as follows: glmmADMB, pscl, stringr, plyr, coda, and lme4. All statistical testing is reported in Table 2.

**Table 2:** Statistical analysis 95% confidence intervals are given for normally distributed data and 5th – 95th percentiles are given for non-parametric data

	Dataset	Data structure	Type of test	95% CI/5th and 95th percentiles
**a**	Blood data: Na	Normal	Linear mixed model	150.69–153.23
**b**	Blood data: HCT	Normal	Linear mixed model	39.95–42.51
**c**	Blood data: Cr	Normal	Linear mixed model	109.38–120.12
**d**	Blood data: urea	Normal	Linear mixed model	6.34–7.14
**e**	Urine data: osmolality	Normal	Linear mixed model	755.29–1476.70
**f**	Urine data: Cr	Normal	Linear mixed model	17.71–41.01
**g**	Urine data: SG	Normal	Linear mixed model	1.022–1.040
**h**	Blood data CfM: urea	Normal	Linear mixed model	7.25–8.39
**i**	Blood data CfM: Cr	Normal	Linear mixed model	91.80–106.44
**j**	Blood data CfM: Na	Normal	Linear mixed model	150.39–153.40
**k**	Blood data CfM: HCT	Normal	Linear mixed model	42.42–45.79
**l**	Percentage weight change: week	Normal	Linear mixed model	−0.78 to 0.033
**m**	Weekly weight change: 5 d	Normal	One-sample *t* test	−1.52 to 0.377
**n**	Weekly weight change: 7 d	Normal	One-sample *t* test	−0.349 to 0.547
**o**	Block weight change: 5 d	Normal	One-sample *t* test	−2.02 to 1.69
**p**	Block weight change: 7 d	Normal	One-sample *t* test	−3.46 to 0.14
**q**	Percentage weight change: block	Normal	Linear mixed model	−2.11 to 0.29
**r**	Weekday behavior: interact	Gamma distribution	ANOVA to compare effects of two linear mixed models	6.84–16.09
**s**	Weekday behavior: locomotion	Gamma distribution	ANOVA to compare effects of two linear mixed models	4.59–7.67
**t**	Weekday behavior: self-groom	Gamma distribution	ANOVA to compare effects of two linear mixed models	3.28–12.20
**u**	Weekday behaviors: body shake	Gamma distribution	ANOVA to compare effects of two linear mixed models	−1.52 to 5.44
**v**	Weekday behaviors: yawn	Gamma distribution	ANOVA to compare effects of two linear mixed models	−5.89 to 11.72
**w**	Weekday behaviors: self-directed	Gamma distribution	ANOVA to compare effects of two linear mixed models	0.025–0.042
**x**	Weekday behaviors: abnormal	Gamma distribution	ANOVA to compare effects of two linear mixed models	1.29–7.07
**y**	Weekday behaviors: social	Zero-inflated	Binary logistic regression	
**z**	Weekday behaviors: inactivity	Poisson	ANOVA to compare effects of two linear mixed models	40.28–61.65
**aa**	Weekday behavior: allogroom	Gamma distribution	ANOVA to compare effects of two linear mixed models	2.82–12.84
**ab**	Weekday behavior: consumption	Gamma distribution	ANOVA to compare effects of two linear mixed models	6.30–19.96
**ac**	Weekday behavior: aggression	Zero inflated	Binary logistic regression	
**ad**	Monkey 3 pacing: weekdays	Non-normal	Kruskal–Wallis	0–78.39
**ae**	Monkey 4 pacing: weekdays	Non-normal	Kruskal–Wallis	23.46–103.13
**af**	Weekend behavior: consumption	Gamma distribution	ANOVA to compare effects of two linear mixed models	1.39–18.19
**ag**	Monkey 3 pacing: weekends	Non-normal	Mann-Whitney	0–50.29
**ah**	Monkey 4 pacing: weekends	Non-normal	Mann-Whitney	20.22–91.83
**ai**	Weekend behavior: interact	Gamma distribution	ANOVA to compare effects of two linear mixed models	6.29–20.42
**aj**	Weekend behavior: locomotion	Gamma distribution	ANOVA to compare effects of two linear mixed models	3.31–7.90
**ak**	Weekend behavior: self-groom	Gamma distribution	ANOVA to compare effects of two linear mixed models	3.40–17.21
**al**	Weekend behavior: inactivity	Poisson	ANOVA to compare effects of two linear mixed models	53.89–87.34
**am**	Weekend behaviors: body shake	Gamma distribution	ANOVA to compare effects of two linear mixed models	−2.07 to 5.16
**an**	Weekend behaviors: yawn	Gamma distribution	ANOVA to compare effects of two linear mixed models	−3.64 to 7.51
**ao**	Weekend behaviors: abnormal	Gamma distribution	ANOVA to compare effects of two linear mixed models	−10.01 to 17.93
**ap**	Weekend behaviors: self-directed	Gamma distribution	ANOVA to compare effects of two linear mixed models	0.019–0.039
**aq**	Weekend behavior: aggression	Zero-inflated	Binary logistic regression	
**ar**	Weekend behaviors: social	Zero-inflated	Binary logistic regression	
**as**	Latency to drink: Saturdays	Non-normal	Mann-Whitney	0.5–9
**at**	Volume consumed	Non-normal	Mann-Whitney	3.51–132.17
**au**	Latency to drink: Sundays	Non-normal	Mann-Whitney	0.5–399.6
**av**	Monkey 2 trial data	Non-normal	Mann-Whitney	152.4–1560.2
**aw**	Monkey 3 trial data	Non-normal	Mann-Whitney	387.55–1280.15
**ax**	Monkey 4 trial data	Non-normal	Mann-Whitney	649–1282.75
**ay**	Water consumption vs Monday trial numbers	Normal	Pearson correlation	
**az**	Weekend weight change	Normal	*t* test	−0.50 to 0.58
**ba**	5 d Monday trial performance	Non-normal	One-sample Wilcoxon signed rank test	154.35–1206.15
**bb**	7 d Monday trial performance	Non-normal	One-sample Wilcoxon signed rank test	167.10–1441.30

#### Physiological data

All data from the physiological measures were normally distributed and analyzed using a linear mixed model (LMM), with fluid control protocol (free access, 5 d, and 7 d) as a fixed factor, and monkey as a random factor. For blood urea and urine osmolality, the variance of the random effect was <0.001, and so the tests were performed with the random effect omitted. To compare blood results from the study to those obtained at the breeding facility, a linear mixed model was used, with monkey colony as a fixed factor and monkey as a random factor.

#### Weight data

We assessed weight loss in the following three ways: over a working week, over each 4 week fluid control protocol, and over a weekend. Weight change over the working week (Monday to Friday) was calculated in the following way: (weight in kilograms Friday/weight in kilograms Monday) − 1*100. The weight changes for the 5 d and 7 d protocols were normally distributed and were compared using a linear mixed model with the percentage of weight change as the fixed factor and monkey as the random factor. This allowed short-term weight change to be assessed. Additionally for each fluid control, weekly weight changes were compared to zero (no change in weight) using a one-sample *t* test. Overall weight change for a fluid control block (4 weeks) was evaluated by taking the start and end weights of the animals and calculating the percentage weight change. Finally, to assess the changes in weights over the weekend, the percentage weight change from Friday to Monday was calculated, and results from the two protocols were compared using a *t* test.

#### Behavioral data

To increase the power of analyses and to detect potentially subtle changes among fluid control protocols, behaviors with similar functions (e.g., foraging, chewing, and eating) were grouped together and analyzed in categories (Table 3). We first tested whether there was a difference in specific behaviors across the three conditions (free access, 5 d, and 7 d). Where there were differences among the three conditions, we investigated whether the 5 d and 7 d protocols differed from the free access data, and from each other. Certain behaviors were never seen and could not be analyzed. These were rocking, head flicking, hand shake, self-injurious behavior, attack, and “other” behaviors (noteworthy behaviors not defined in the ethogram, see Table 1).

**Table 3:** Categories of behaviors used for statistical analysis

Category	Included behaviors
Scan sampled (every 30 s)	
Inactivity	Alert, not alert, hunched
Consumption	Eating, chewing, foraging
Interact	Interact with physical environment–hands/feet; interact with physical environment–oral
Locomotion	Relaxed locomotion, agitated locomotion
Allogroom	Allogroom–donor; allogroom–recipient
Self-groom	Self-groom
Pacing	Locomotor stereotypy
Continuously sampled (duration)	
Aggression	Aggression to cage mate, aggression directed outside cage
Social	Affiliative, being mounted, dominance, mounting, play with cage mate, submissive to cage mate
Self-directed	Self-scratching, eye rub
Continuously sampled (frequency)	
Body shake	Body shake
Yawn	Yawn
Abnormal	Other abnormal

Continuously sampled behaviors occurred infrequently in the 30 s scan samples because of their rare or short nature and were therefore omitted from the scan sample data and analyzed separately. As described above, drinking behavior was captured separately as the latency to approach the bottle and the volume consumed in 5 min at weekend time points. Since animals were separated from their cage mate on Saturdays, behavioral repertoires were not directly comparable between weekdays and weekends. Therefore, separate analyses were performed for weekday data and Saturday data.

Behaviors were analyzed by creating two models in R. The first was a linear mixed model with an underlying gamma distribution, with monkey identity as a random factor and fluid control as a fixed factor. A second model omitting the effect of fluid control was created and an ANOVA was applied to compare the two models, to assess the overall main effect of fluid control (Crawley, 2005). Scan-sampled behaviors (excluding inactivity and pacing), all continuously sampled frequency behaviors, and self-directed behavior were analyzed in this way. Inactivity was also fitted to the above models using an underlying Poisson distribution.

Some behaviors occurred at low frequency or were not performed by all animals and, so, were analyzed separately. Pacing was performed by only two individuals and did not follow a normal distribution. It was therefore analyzed separately for each animal using a Kruskal–Wallis test for weekdays and Mann–Whitney *U* test for Saturdays. Due to the low occurrence of social behavior and aggression, and the high prevalence of zeros in the data, these two categories were analyzed using a binary logistic regression, with a random factor of monkey identity and fluid control as a fixed factor.

#### Water bottle approach and consumption

Latencies to approach the bottles were not normally distributed and were analyzed by using a Mann–Whitney *U* test. In order to make Saturday consumption data comparable across the monkeys, volumes drunk were converted to a percentage of the minimum daily allowance for each animal. These data were not normally distributed and were analyzed using a Mann–Whitney *U* test.

#### Task performance

Monkey 1 was excluded from the task performance analysis (i.e., the number of trials performed on workdays as a function of fluid control protocol). This was due to the difficulty of his task increasing across the study, as was necessary for the electrophysiological data collection, and the varied setting in which he worked (electrophysiology laboratory and MRI scanner). Trial data for the remaining three monkeys were not normally distributed, and differences in the number of trials performed when on the 5 and 7 d fluid control protocols were assessed using a Mann–Whitney *U* test for each monkey individually. To assess the effect of weekend water intake on Monday work performance, a Pearson correlation was calculated using the percentage weight change from Friday to Monday and the number of trials performed on a Monday. In addition, trials performed on Monday were compared to 1000 (an approximately acceptable laboratory performance) using a one-sample Wilcoxon sign rank test.

## Results

### Fluid intake of individual animals

The four animals differed in their FAI and in the minima established (see Materials and Methods) to ensure adequate work rates, as follows: Monkey 1, 645 ml FAI, minimum 200 ml (31% FAI or 15 ml/kg/d); Monkey 2, 880 ml FAI, minimum 150 ml (17% FAI or 14 ml/kg/d); Monkey 3, 910 ml FAI, 355 ml minimum (39% FAI or 26 ml/kg/d); and Monkey 4, 305 ml FAI, minimum 150 ml (49% FAI or 17 ml/kg/d).

### Physiological measures

**Figure 1.**
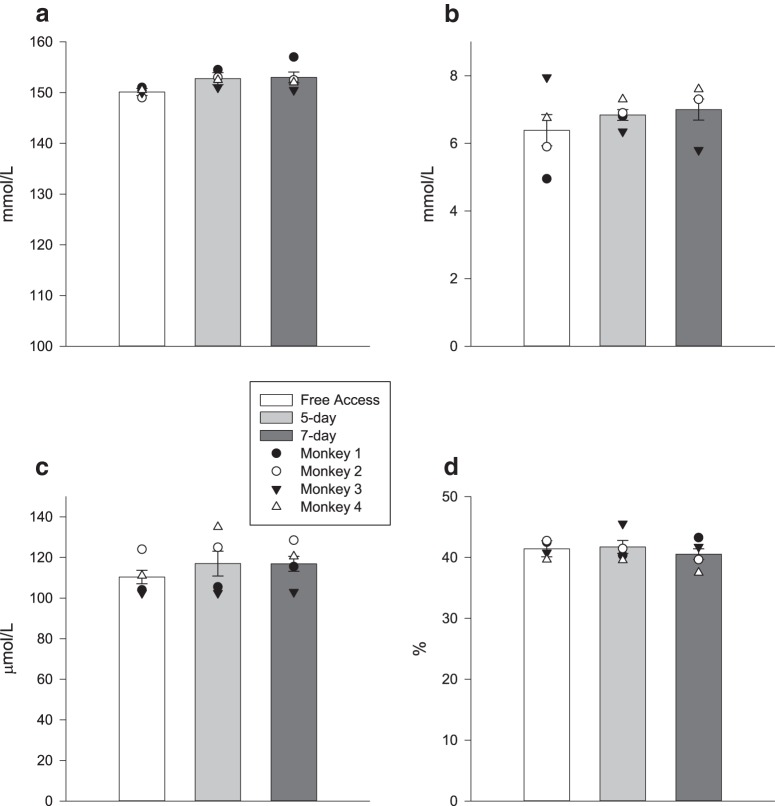
***a–d***, The effect of fluid restriction protocols on blood measures of hydration (mean ± SEM): sodium (***a***); urea (***b***); creatinine (***c***); and hematocrit (***d***). The means for individual monkeys are denoted by the overlaid symbols.

We found that concentrations of sodium (Na), hematocrit (HCT), urea, and creatinine (Cr) in the blood did not significantly differ between the 5 d protocol and the 7 d protocol, and the free access period (LMM: Na, HCT, Cr: *F*_(2,18)_ < 2.98, *p* > 0.076^a-c^; urea: *F*_(2,21)_ = 0.894, *p* = 0.424^d^; Fig. 1). Urine Osm, Cr, and specific gravity (SG) significantly differed across conditions (LMM: Osm: *F*_(2,11)_ = 16.91, *p* < 0.001^e^; Cr: *F*_(2, 9.98)_ = 7.32, *p* = 0.011^f^; SG: *F*_(2,9.58)_ = 24.30, *p* < 0.001^g^). All three urine measures were lower when monkeys had free access to water than during either the 5 d or the 7 d protocol (Bonferroni *post hoc* comparisons, all *p* < 0.05; Table 4), but there was no difference between the two protocols (Bonferroni *post hoc* comparisons, all *p* > 0.54; Table 4).

**Table 4:** The effect of fluid control on urine measures of hydration Bonferonni *post hoc* pairwise comparisons between the different fluid control conditions (Free access, 5-d and 7-d protocol)

Measure	(I) Fluid control	(J) Fluid control	Mean difference (I–J)	SEM	df	*p* Value
Osmolality (mOsmol/kg)	Free access	5 d	−1205.2	143.46	11	<0.001
		7 d	−865.1	151.66	11	0.008
	5 d	7 d	340.1	154.82	11	0.48
Creatinine (mmol/L)	Free access	5 d	−28.41	8.97	10.16	0.03
		7 d	−30.60	8.90	9.62	0.02
	5 d	7 d	−2.19	8.97	10.16	1.00
SG	Free access	5 d	−0.03	0.005	9.71	<0.001
		7 d	−0.03	0.005	9.33	0.001
	5 d	7 d	0.006	0.005	9.71	0.66

We found that the levels of blood urea were higher at the CfM breeding center (mean difference: 1.08 mmol/L, *F*_(1,40)_ = 8.36, *p* = 0.006^h^). Creatinine levels were also lower at the CfM (mean difference: 15.75 μmol/L, *F*_(1,11.08)_ = 5.79, *p* = 0.035^i^). The remaining blood measures were not significantly different between colonies (Na: *F*_(1,12.99)_ = 0.004, *p* = 0.95^j^; HCT: *F*_(1,8.78)_ = 4.60; *p* = 0.06^k^).

### Weights

**Figure 2.**
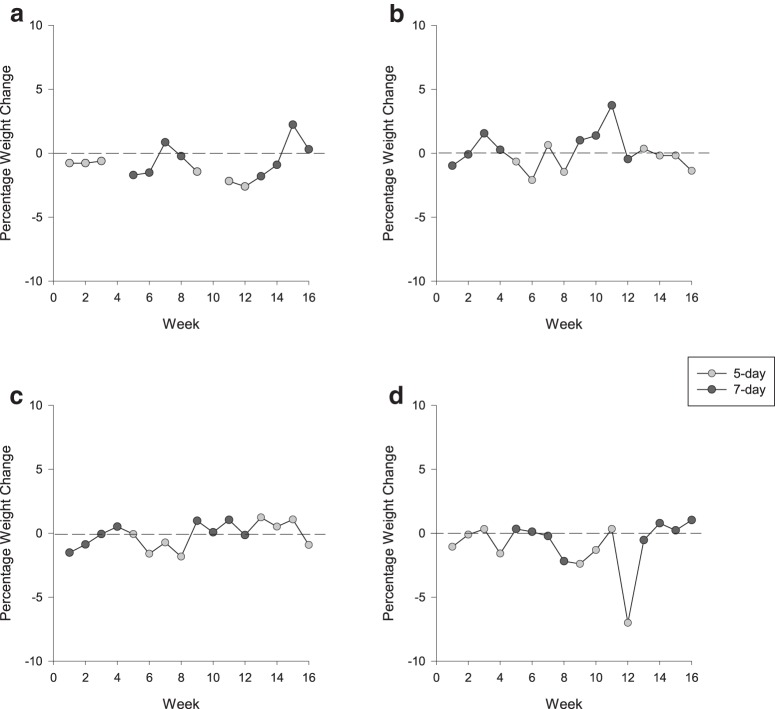
***a–d***, The weekly percentage weight change calculated from the beginning of each fluid control block ([weight in kilograms Friday/weight in kilograms Monday] − 1*100): Monkey 1 (***a***); Monkey 2 (***b***); Monkey 3 (***c***); and Monkey 4 (***d***). Dashed lines indicate no change in weight.

To investigate weight loss, we measured daily changes in body weight across the working week, across the 16 weeks of fluid control. From Monday to Friday, weight loss occurred on the 5 d protocol but not on the 7 d protocol (LMM: *F*_(1,57.20)_ = 9.48, *p* = 0.003^l^; Fig. 2). On average, monkeys lost body mass (mean weight change, −0.95%) on the 5 d protocol (one-sample *t* test, test value = 0, *t*_(29)_ = 3.39 *p* = 0.002^m^), while their body mass remained relatively constant (mean weight change, +0.10%) on the 7 d protocol (*t*_(31)_ = 0.452, *p* = 0.655^n^). However, across a fluid control block (4 weeks), we found the opposite trend, with animals maintaining weight on the 5 d protocol (mean weight change, −0.16%), but losing weight on the 7 d protocol (mean weight change, −1.66%). However, neither change was significantly different from zero (one-sample *t* test, test value = 0, 5 d protocol: *t*_(7)_ = 0.208, *p* = 0.814^o^; 7 d protocol: *t*_(7)_ = 2.18, *p* = 0.066^p^), nor from one another (LMM: *F*_(1,11)_ = 2.12, *p* = 0.173^q^).

### Behavioral measures

We first tested whether there was a difference in specific behaviors across the three conditions (free access, 5 d, and 7 d). Where there were differences among the three conditions, we then investigated whether the 5 d and 7 d protocols differed from the free access data, and from each other.

#### Weekdays

**Figure 3.**
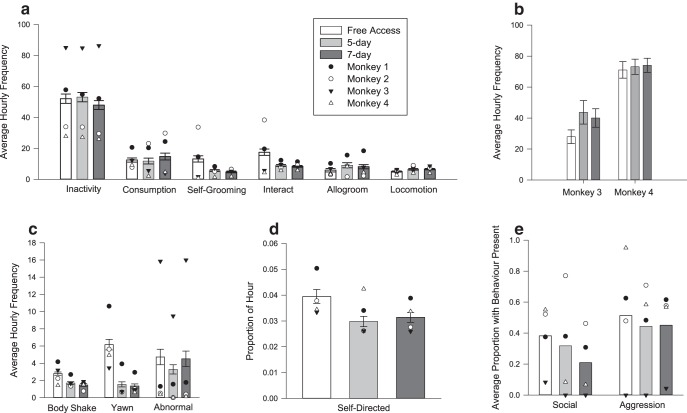
***a–e***, The effect of free access to water, and 5 d and 7 d fluid control protocols on behaviors performed on weekdays. Behaviors are grouped by the sampling methods used (see Materials and Methods), as follows: scan-sampled behaviors (***a***); scan-sampled pacing frequency for Monkeys 3 and 4 (***b***); continuously sampled, frequency-only behaviors (***c***); continuously sampled duration of self-directed behavior (***d***); and continuously sampled behaviors (binary data) with a high prevalence of zeros (***e***). The means for individual monkeys are denoted by the overlaid symbols.

Significant differences in the frequency of nine behaviors occurred across the free access and fluid control protocols, as follows: interaction (χ^2^ (2) = 42.27, *p* < 0.001^r^); locomotion (χ^2^ (2) = 11.77, *p* = 0.0027^s^); self-groom (χ^2^ (2) = 37.35, *p* < 0.001^t^); body shake (χ^2^ (2) = 30.86, *p* < 0.001^u^); yawn (χ^2^ (2) = 101.32, *p* < 0.001^v^); self-directed (χ^2^ (2) = 17.09, *p* < 0.001^w^); abnormal (χ^2^ (2) = 10.07, *p* = 0.0065^x^); social (χ^2^ (2) = 8.72, *p* = 0.013^y^); and inactivity (χ^2^ (2) = 6.51, *p* = 0.039^z^; Fig. 3*a–e*). For six of these behaviors (interaction, locomotion, self-groom, body shake, yawn, and self-directed), the frequency was lower in the 5 d and 7 d protocols compared with free access (5 d protocol: all *t*_(184)_ < 7.06, *p* < 0.006; 7 d protocol: all *t*_(194)_ < 7.69, *p* < 0.001), with no difference in frequency between the two fluid control protocols (all *t*_(198)_ < 1.07, *p* > 0.28).

Three of 13 behavioral categories differed between the two fluid control protocols. Abnormal behavior was lower in frequency in the 5 d protocol compared with free access (*t*_(184)_ = 2.68, *p* < 0.001) and the 7 d protocol (*t*_(198)_ = 2.79, *p* = 0.005), but there was no difference between free access and the 7 d protocol (*t*_(194)_ = 0.08, *p* = 0.940). However, inactivity was lower on the 7 d protocol compared with free access (*t*_(194)_ = 2.55, *p* = 0.01), but was not different from the 5 d protocol (*t*_(198)_ = 1.39, *p* = 0.166). There was also no difference between free access and the 5 d protocol (*t*_(184)_ = 1.18, *p* = 0.237). Social behavior was lower on the 7 d protocol than on the 5 d protocol (*t*_(198)_ = 2.13, *p* = 0.033) and free access (*t*_(194)_ = 0.2.79, *p* = 0.005), but there was no difference between free access and the 5 d protocol (*t*_(184)_ = 0.76, *p* = 0.45).

No other behaviors were affected by fluid control (allogroom and consumption (foraging, eating, and chewing): χ^2^ (2) < 2.99; aggression, χ^2^ (2) = 1.08; pacing, H_2_ < 3.36; all *p* > 0.16^aa-ae^; Fig. 3*a*,*b*,*e*).

#### Weekend

**Figure 4.**
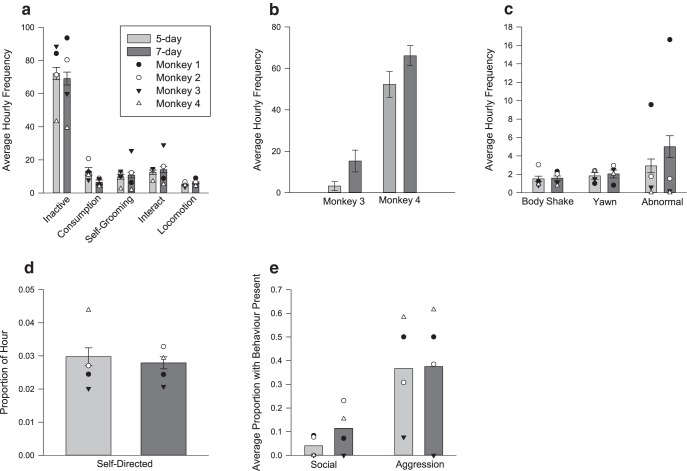
***a–e***, The effect of 5 d and 7 d fluid control protocols on behaviors performed on Saturdays. Behaviors are grouped by the sampling methods used (see Materials and Methods), as follows: scan-sampled behaviors (***a***); scan-sampled pacing frequency for Monkeys 3 and 4 (***b***); continuously sampled, frequency-only behaviors (***c***); continuously sampled duration of self-directed behavior (***d***); and continuously sampled behaviors (binary data) with a high prevalence of zeros (***e***). The means for individual monkeys are denoted by the overlaid symbols.

We found a significant effect of fluid control on two behaviors on weekends. The first was consumption (foraging, chewing and eating), which was lower when animals were on the 7 d protocol compared with the 5 d protocol (χ^2^ (1) = 8.68, *p* = 0.003^af^). The second behavior was pacing, which was only sufficiently frequent to allow for quantitative analysis in two of the four animals. We found that pacing increased for one monkey on the 7 d protocol compared with the 5 d protocol (*U* = 110, *z* = 2.43, *p* = 0.026^ag^; Fig. 4*b*), while the second monkey showed no change in pacing behavior (*U* = 107, *z* = 1.58, *p* = 0.123^ah^; Fig. 4*b*). All remaining behaviors showed no difference in frequency between the 5 d and 7 d protocols (interaction, locomotion, self-groom, inactivity, body shake, yawn, abnormal, self-directed, aggression, and social: χ^2^ (1) < 3.23, *p* > 0.07^ai-ar^ for all; Fig. 4*a*,*c*,*d*,*e*).

#### Water bottle approach and consumption

On Saturdays, approach was significantly quicker on the 7 d protocol (median time, 2 s), than on the 5 d protocol (median time, 4 s; Mann–Whitney test: *U* = 2.24, *p* = 0.03^as^). The monkeys also drank more in 5 min on Saturdays while on the 7 d protocol (median percentage of the minimum amount consumed, 100%), compared with while on the 5 d protocol (median percentage of the minimum consumed, 50%; Mann–Whitney test: *U* = 3.28, *p* = 0.001^at^). There was no effect of fluid control protocol on the latency to approach the water bottle on Sundays (Mann–Whitney test: *U* = 0.46, *p* = 0.647^au^).

### Task performance

**Figure 5.**
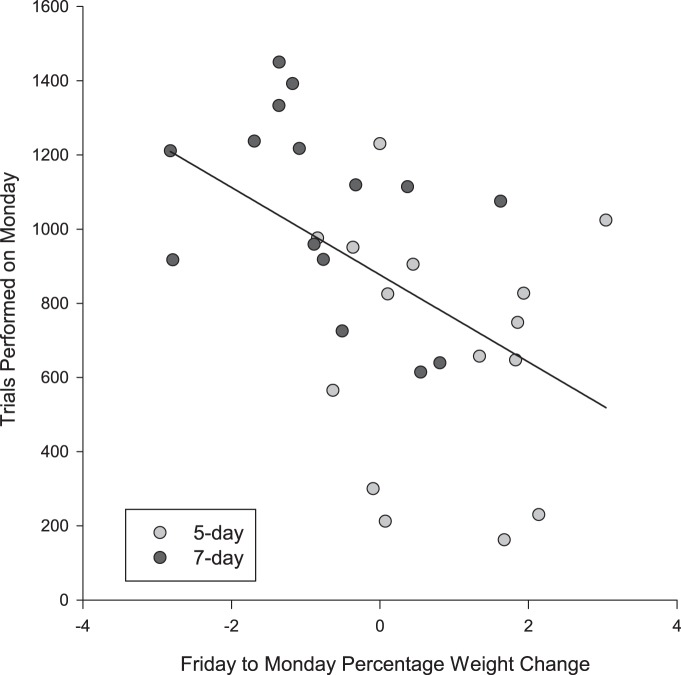
The effect of the percentage weight change from Friday to Monday on the number of trials performed on a Monday.

Only three monkeys were included in the analysis (one monkey had regularly changing task demands that were required by the experimental design, which precluded this specific analysis). There was no overall increase in the daily numbers of trials performed in their respective cognitive tasks when they were subjected to the 7 d protocol, rather than the 5 d protocol (Mann–Whitney test: *U* < 1.44, *p* > 0.15 for all^av-ax^). The performance on Monday is of particular importance, since animals often do not perform enough trials for scientific data to be collected. On Mondays, there was a significant correlation between the percentage weight change over the weekend (from Friday to Monday) and the number of trials performed: when weight decreased over the weekend, more trials were performed on the Monday (Pearson correlation: *R* ^2^ = −0.49, *p* < 0.01^ay^; Fig. 5). Weight change over the weekend differed between the two fluid controls (*t* test: *t*_(28)_ = 3.58, *p* = 0.001^az^). On average, monkeys gained 0.83% weight over the weekend on the 5 d fluid control and performed <1000 trials on Mondays (median number of trials, 686; test value, 1000; W = 3.64, *p* < 0.001^ba^). Conversely, on the 7 d protocol, monkeys lost 0.76% of body mass and completed an average of 981 trials on Mondays (test value, 1000, W = 0.065, *p* = 0.948^bb^).

## Discussion

Our study provides the first objective and quantitative data on the effects of fluid control protocols on the physiology, behavior, and performance of laboratory macaques used in behavioral neuroscience. Given the range of data analyzed, we discuss each of our different measures in turn.

### Physiological changes

One primary concern with fluid control protocols is that they dehydrate the animals, leading to reduced welfare and poor animal condition (Prescott et al., 2010). We took standard and well established physiological measures of hydration state for rhesus macaques using blood and urine (Rolls and Rolls, 1982; Wood et al., 1982). For these measures, “normal” ranges from non-fluid-controlled individuals have been reported from various rhesus macaque facilities (Levine, 1995; Buchl and Howard, 1997; Hassimoto et al., 2004; Ribeiro Andrade et al., 2004; Chen et al., 2009; Lee et al., 2012; National Institute on Aging and National Primate Research Center, 2015; Table 5). Blood measures of hydration were the same across all three conditions (5 d protocol, 7 d protocol, and free access), and levels were within ranges observed across other rhesus macaque facilities where animals have constant *ad libitum* fluid access (Table 5). Urine was more concentrated on both fluid control protocols compared with free access, although there were no differences between the two fluid control protocols. However, since the values in Table 5 refer to populations including animals of differing ages compared with our males, we also compared the blood measures with blood that we acquired from the CfM, from a sample of similarly aged male monkeys. Males at the CfM had never experienced any fluid control protocol. Two blood measures did differ slightly between our monkeys and those at the CfM: the CfM macaques had higher levels of urea and lower levels of creatinine. The higher levels of urea were the opposite of what we would expect for animals with *ad libitum* access to water. Although the reason for these differences is unknown, values for both colonies still lie within normal ranges (Table 5). Overall, the monkeys’ kidneys were well functioning and efficiently retaining fluids when access to water was limited. Their ability to efficiently retain fluids may be an adaptation to seasonal rainfall and periods of restricted water access in their natural environment (Lindburg, 1977). It is important to highlight that all four macaques used in this study had been previously water restricted on the 5 d protocol for >4 years, and their physiological responses to fluid control remained normal. Thus, the 5 d protocol as used has no negative physiological effects on a long-term basis (>4 years), refuting the idea that keeping animals on such fluid control protocols for long periods may cause physiological harm (Prescott et al., 2010).

**Table 5:** Values of normal physiological hydration measures Published blood parameter ranges for macaques given free access to water

Source	Age (years)	*N*	Na (mmol/L)		Urea (mmol/L)		Creatinine (µmol/L)		HCT (%)	
			Mean	SD	Mean	SD	Mean	SD	Mean	SD
Lee et al., 2012	2-5	29	145.68	3.68	5.91	1.59	66.3	15.91	34.87	4.49
Hassimoto et al., 2004	3.5	6	139	5	9.28	1.43	41.55	6.19	43.4	4.7
Chen et al., 2009	3-5	18	149.71	3.07	8.47	1.21	69.73	11.51	43	0.02
Ribeiro Andrade et al., 2004	3.5-16	21			11.13	3.71			37.55	3.23
Primate aging database (indoor housing)	4-15	57- 157	148.88	11.48	6.40	2.98	111.65	71.07	41.04	10.35
Primate aging database (indoor housing)	8-9	3 - 44	150.33	1.7	6.14	2.01	122.35	18.39	40.42	3.58
Primate aging database (All housing)	4-15	62 - 192	146.382	9.00	6.81	3.38	109.09	67.80	41.04	9.88
Primate aging database (all housing)	8-9	15 - 53	145.5	8.55	6.86	2.59	112.62	20.69	40.62	3.42
Buchl and Howard (1997)	3-4	30	148	3	6.43	1.07	79.46	8.84		
Levine (1995)	3-7		145	1.5	7.14	1.07	83.98	11.05		

Overall, our physiological measures suggest that there is no short-term welfare impact from being on either protocol over a 4 week period, and no significant difference between the two. While our data also suggest no long-term (>4 years) harm caused by monkeys being repeatedly subject to the 5 d protocol for fluid control, we cannot be certain that this is the case for the 7 d protocol for fluid control, as the 7 d fluid control regime has not been implemented for extended periods of time. Further long-term studies would be required to investigate this.

### Weight change

Potential weight loss is a key welfare issue surrounding fluid control, with concerns that fluid control and the potential associated reduction in food intake (Cizek and Nocenti 1965; Collier and Levitsky 1967; Engell 1988) could lead to a substantial loss in body mass (Prescott et al., 2010). Within a working week (Monday-Friday), weight loss occurred on the 5 d protocol but not on the 7 d protocol. However, across a fluid control block, we found the opposite effect, with animals maintaining weight over the 5 d protocol, but not over the 7 d protocol, where there was a small degree of weight loss (∼2% over a 4 week period). Although these results initially appear contradictory, they can be explained by weight changes over the weekend. When on the 5 d protocol, monkeys tended to gain weight when they had free access to water, thus starting the week at a higher weight (mean weight change, +0.83%). In contrast, without the opportunity to work beyond their minimum on weekend days, monkeys on the 7 d protocol tended to lose weight over a weekend (mean weight change, −0.76%), resulting in a slight weight loss over the 4 week block. Whether weight loss would continue on an extended 7 d protocol is impossible to say, given the results of our study, and would require further longer-term research. However, our data are conclusive in showing that a 5 d protocol does not lead to excessive weight loss or, indeed, to any weight loss, and a 7 d restriction regime over the course of 4 weeks induces no statistically significant weight loss or any rapid or sustained weight loss that would raise any immediate welfare concern.

### Behavior

There were some behavioral changes in our monkeys between the free access and fluid control conditions. While some behavioral changes may be indicative of reduced welfare during the two fluid control protocols, for example, increased stereotypic pacing in one animal (Gottlieb et al., 2015), others suggest the opposite: that the welfare of the monkeys was compromised more during the control period. Body shaking, self-grooming, and yawning are considered to be indicative of anxiety in macaques (Ninan et al. 1982; Deputte 1994; Schino et al. 1996; Major et al., 2009), making it surprising that these behaviors were more prevalent during the free access period compared with during either fluid control protocol. One possible reason for this observation was that the data on free access were collected over the Christmas break, when animals were not working in experiments and had free access to water. Collecting data on free access during breaks was necessary because fluid control and working routines are intrinsically linked. Fluid control is only permitted when the monkeys have the opportunity to earn fluid in the laboratory, and running animals in experiments on free access is not possible. However, this meant that there were also changes to laboratory and husbandry routines: monkeys did not take part in experimental procedures and had reduced social contact with humans (research and animal care staff); and husbandry routines were different from those experienced during a typical experimental week. Although animals may experience similar periods throughout the year (e.g., holiday weekends, and festive breaks), these changes in routine could potentially increase anxiety-related behaviors in the free access period (for review, see Bassett and Buchanan-Smith, 2007). Therefore, it is difficult to know whether behavioral differences between free access and fluid control protocols were due to fluid access, changes in routine, or a combination of the two. When husbandry and daily routine return to normal, the corresponding decrease in anxiety could theoretically mask an increase in anxiety from fluid restriction. Despite this potential confound, we can safely conclude that fluid control does not increase anxiety more than a change in husbandry regime.

There were also very few behavioral differences observed between the two fluid control protocols, and again, the results were inconsistent. For example, on weekdays while monkeys were on the 7 d protocol for fluid control, rare social behaviors (not including allogrooming) were lower and abnormal behaviors were higher, which is sometimes indicative of increased stress (Lutz et al., 2003; Honess et al., 2004; Smith et al., 2006). However, in contrast to this, on the 7 d protocol inactivity was lower, which normally demonstrates improved welfare. It is surprising that inactivity decreased in monkeys subjected to a stricter fluid control, since studies on humans (Pross et al., 2014) have documented an increase in fatigue when subjects are fluid deprived, with participants anecdotally reporting decreased activity levels. Decreasing activity levels are inconsistent with the observed increase in abnormal behaviors, making it impossible to identify any clear impacts on welfare from the 7 d protocol.

Small behavioral differences between protocols were also observed on Saturdays. Consumption (foraging, eating, and chewing) was lower on the 7 d protocol compared with the 5 d protocol. There are two possible explanations for this. One possible explanation is that because water is required to absorb and digest food, animals cannot eat as much on the 7 d protocol compared with the 5 d protocol. This voluntary reduction in consumption has been previously documented in rats and humans (Cizek and Nocenti, 1965; Collier and Levitsky, 1967; Engell, 1988) and is one of the concerns surrounding fluid control (Prescott et al., 2010). Alternatively, it may not be that the animals are undereating on the 7 d protocol, but rather that they are overeating on the 5 d protocol: “binging” can occur when monkeys are given free access to water on the 5 d regime (Toth and Gardiner, 2000). Both of these explanations are supported by changes in weight over the weekend (see above), with increases on the 5 d protocol but decreases on the 7 d protocol, making it difficult to tease apart the two. Overall, regardless of what causes the difference in consumption behavior on weekends, these changes were not of a magnitude to cause weight loss that was of concern in our monkeys.

The second change was in pacing behavior. Two of the animals in our group performed pacing behavior during all protocols. In one of the two, higher levels of pacing occurred over the weekend on the 7 d protocol compared with the 5 d protocol. Stereotypies in captive macaques are often used as indicators of suboptimal welfare and may indicate higher levels of stress in this individual (Novak et al., 2006). However, their prevalence alone should not be relied upon as a single measure of well-being (Mason and Latham, 2004), and data from one animal remain too limited to enable a firm conclusion. In addition, stereotypies can be interpreted as a coping behavior (Mason and Latham, 2004; Novak et al., 2006), and, as such, the animals performing these behaviors may experience a more positive state of well-being than is often assumed.

### Water bottle approach and consumption

When given access to water on Saturdays, monkeys appeared more motivated to drink on the 7 d protocol than on the 5 d protocol. They approached the bottle more quickly and consumed a larger volume of water. This may be due to many reasons, including a dryness or unpleasant taste in the mouth, as has been shown in humans (Rolls et al., 1980). However, it is impossible to infer the subjective experience (e.g., thirst) of the animals from our data.

### Task performance

An important aspect of this study was to assess the scientific outcomes associated with the use of different fluid control protocols. Typically, on a 5 d fluid control regime, animals do not participate in a sufficient number of trials on Monday to collect a robust dataset (∼1000 trials are required per day for these particular tasks). Consequently, data collection is not usually attempted on a Monday. The number of trials performed on a Monday in this study was too low on the 5 d fluid control protocol to attempt electrophysiological recordings, given the scientific requirements of the studies involved. The most likely reason for this is that monkeys were not motivated to drink after increased access to water over the weekend. However, when the monkeys were restricted over the weekend on the 7 d protocol, performance on Mondays increased to levels that would generally allow electrophysiology to be performed. This suggests that a 7 d fluid control protocol might enable scientific studies to be conducted 5 d/week (or 7 d/week, if recording continued over the weekend), which could significantly reduce the duration of a study by at least 20%. This would mean that the time individual monkeys spend on a fluid control protocol would be similarly reduced.

### Conclusions

As with any procedure, the consequences of fluid control on the state of the subject are important to know so that judgments about the appropriateness for their use can be made on knowing the likely impact on the results. This study addressed the need for scientific data on the impact of different fluid control protocols on the welfare and performance of laboratory primates used in neuroscience research (Prescott et al. 2010). The debate over the use of fluid control protocols has been contentious (Orlans, 1991; Willems, 2009; Westlund, 2012), and it is crucial that we better understand how such protocols affect experimental animals in order to make more informed decisions about their use. Our main conclusions are as follows:

1. Male macaques physiologically cope with periods of fluid control, maintaining blood parameters within normal ranges by concentrating their urine in response to both protocols. There were no detectable short-term effects of either the 5 d or 7 d protocol, or any long-term (>4 years) effect of a 5 d protocol, on kidney function.
2. There were relatively small changes in behavior detected by our in-depth analysis, with some behaviors indicative of poor welfare being associated with fluid control protocols, and others with free access to water.
3. 5 d and 7 d fluid control protocols do not lead to rapid and sustained weight loss that would be of immediate welfare concern. More data are required to assess the continuing impact of 7 d fluid control on weight changes.
4. Animals are more motivated to drink in their home cage when on a 7 d protocol compared with a 5 d protocol, but the subjective experiences of the animals are unknown.
5. Improved task performance on a 7 d protocol compared with a 5 d protocol could allow more rapid collection of sufficient scientific data and a reduced amount of time spent on fluid control protocols for experimental animals.

Our data mostly fail to show the significant detrimental effects on the welfare of laboratory macaques, which often have been predicted to arise from the use of fluid control protocols. Our study counters some and alleviates many of the widely held welfare concerns surrounding these methods.

## Synthesis

The decision was a result of the Reviewing Editor Philippe Tobler and the peer reviewers coming together and discussing their recommendations until a consensus was reached. A fact-based synthesis statement explaining their decision and outlining what is needed to prepare a revision is listed below. The following reviewers agreed to reveal their identity: Michele Basso, Barry Richmond

Two referees have seen the manuscript. They found it greatly improved and believe that it now will make a nice contribution. Sticking to the traditional format, including putting the Methods before the Results, makes for a more easily read and understood manuscript.

Points:

1. With the small sample size it really is important that the data points for all 4 subjects be superimposed on the data figures. I agree that the authors have done a really strong job on the statistics, but 4 samples is really small, and I cannot figure out why the authors resist doing this - it does not make a good impression. In this day and age of computer graphing, adding the data points is no more that a procedural annoyance for the authors. Add them.

2. One possible explanation for the higher BUN in the control colony group might be a higher protein diet. BUN is sensitive to the protein content of the diet. The reason that creatinine is used for assessing kidney function is that it is not very sensitive to protein in the diet, but rather kidney function. BUN is sensitive to both. A last possibility is sampling problems from the small size of the experimental - 4 animals that just happen to constitutionally have BUNs that tend to the high end of the normal range.

Some little points:

Line 5 - don't need to say 'poorly understood'. Why would you study the topic if it were well understood. Ugly phrase, and the next sentence makes clear what you are doing.

Line 73 - referent for 'none' is not clear - none of what?

Line 388 - '…first…' Is it? You cite others who tried. A bit self-serving.

Lines 414-416 - Do you need to say the mechanisms are still unclear? If you knew we presume you would have said something about them.

Line 417 - '…yet…' Are you surprised? After all you had used them for 4 years, and I presume that seemed in good health over that period.

Line 420 - I think you are trying to say: Our study seems to show no long terms negative consequences from the period on the fluid restriction protocol.

Line 422-428 - Repetitious. Could drop.

Line 430 - Are you trying to say: If the fluid restriction led to a significant decrease food intake, the decrease in food intake might lead to chronic weight loss.

Line 439 - Everywhere else you refer to weight not mass. Probably change to mass -> weight.

Line 443 - what is 'it'? Referent not clear.

Line 481 - 'conflicts' -> 'seems inconsistent' seems better to me.

Line 495-496 - drop 'It should be noted that…' we assume you wrote the rest of the sentence so we would 'note it'. The last half of the sentence is really awkward, rewrite?

Line 502-503 - Why even bring this up? There are only the data from one or two observations in one animal. Not 'rigorous'.

Line 516 - add 'on Monday', otherwise not clear.

Line 531 - drop 'urgent'. For some of us this study is nice to have but the need has not been seen as 'urgent'.

Line 533 - drop 'highly'. How much more is 'highly contentious' than 'contentious'. Again more of a problem in Europe and UK than US or far east. Nonetheless, the data are welcome.

Line 534 and onward - I would like to see something more like: As with any procedure, the consequences of fluid control on the state of the subject are important to know so that judgments about the appropriateness for their use can be made on knowing the likely impact on the results.
